# Characterization of Human Papillomavirus Subtype 72b

**DOI:** 10.1128/genomeA.01320-14

**Published:** 2014-12-18

**Authors:** Hanna Johansson, Evgenia Kravtchenko, Ola Forslund

**Affiliations:** Department of Laboratory Medicine, Medical Microbiology, Lund University, Malmö, Sweden

## Abstract

We report the characterization of human papillomavirus (HPV) subtype 72b of the genus *Alphapapillomavirus* isolated from an oral rinse sample of a healthy woman. The HPV72b L1 open reading frame (ORF) was 90.2% identical to that of HPV72, indicating a subtype close to the border of a novel HPV type.

## GENOME ANNOUNCEMENT

In an oral rinse sample from a 44-year-old woman we identified a novel sub-genomic Forslund Antonsson primer (FAP)-fragment, FA171, with closest sequence identity to HPV72 (1). After cloning of its complete genome we determined that the L1 open reading frame (ORF) was 90.2% identical to that of HPV72 (genus *Alphapapillomavirus*). Therefore, it was labeled as subtype HPV72b of HPV72, originally isolated from an oral papillomatous lesion of an HIV positive patient (2).

The complete genome of HPV72b (8,098 bp) was obtained using the PrimeSTAR GXL DNA polymerase kit (TaKaRa Bio, Shiga, Japan). Briefly, 2.5 µL purified DNA (Magna LC, Roche) was amplified in 25 µL containing a 1× PrimeSTAR GXL buffer, 200 µM of each dNTP, 0.2 µM of each primer (fwd 5′-TGA CTA CAA GCA AAC ACA GTT GCT T-3′ and rev 5′-ACA GAT ATA TTG TCC CGG CTG TC-3′ from DNA-Technology, Denmark), and 0.625 U PrimeSTAR GXL DNA polymerase. Amplification was performed for 45 cycles at 98°C for 10 s, 60°C for 15 s, and 68°C for 8 min in a thermocycler (Mastercycler Eppendorf, Germany). The amplicon was cloned using the TOPO TA cloning kit and the pCR 2.1-TOPO vector (Invitrogen, Carlsbad, CA) and sequenced using primer-walking (Eurofins, Germany).

A novel HPV type shares less than 90% similarity to the closest papillomavirus (PV) type in the L1 ORF (3). Pairwise comparisons between L1 ORFs of HPV72b and HPV72 (second ATG) (2) demonstrated 90.2% nucleotide (nt) identity, whereas the other ORFs showed identities less than 90% (E6: 85.0%, E7: 85.4%, E1: 89.2%, E2: 85.5%, E4: 80.6%, E5: 69.5%, L2: 87.2%) and URR 78.6%. The overall similarity between HPV72b and HPV72 was 85.3%. There was also a segment of 101 nt (3,874 to 3,974) downstream of the HPV72b E2 ORF that was not present in HPV72.

HPV72b had a G+C content of 45.3% and the typical genome organization of HPVs of the genus *Alphapapillomavirus* species-3 with an E5-beta ORF (nt 4,241 to 4,369) (4). In the upstream regulatory region (URR) of HPV72b (804 bp) we identified five consensus E2-binding sites (ACC-N_6_-GGT), one putative TATA box (TATAA), and one putative polyadenylation site (ATATAA).

The putative E6 proteins contained two zinc-finger domains (CxxC[x]_29_CxxC) (5) separated by 39 amino acids. One zinc-finger domain was present in the E7 protein. The LxCxE-motif (binding site for the pRB) (6) was not observed in the E7 protein as the cysteine was substituted for a serine in the corresponding site, LxSxE, identical to that of HPV72.

The putative E1 protein did not exhibit the conserved ATP-binding site (GPPDTGKS) (7, 8), instead GPSNTGKS was found. The initiation codon of the putative start of E4 ORF was absent, as the ATG codon was changed to ACG.

### Nucleotide sequence accession number.

The complete genomic sequence of HPV72b is available in GenBank under the accession no. KJ145795.
